# Cost-effectiveness of evolocumab in treatment of heterozygous familial hypercholesterolaemia in Bulgaria: measuring health benefit by effectively treated patient-years*

**DOI:** 10.1080/20016689.2017.1412753

**Published:** 2017-12-22

**Authors:** Borislav Borissov, Michael Urbich, Boryana Georgieva, Svetoslav Tsenov, Guillermo Villa

**Affiliations:** ^a^ Prescriptia EOOD, Sofia, Bulgaria; ^b^ Amgen (Europe) GmbH, Economic Modeling Center of Excellence, Zug, Switzerland; ^c^ Amgen Bulgaria EOOD, Value, Access & Policy, Sofia, Bulgaria

**Keywords:** Cardiovascular diseases, hypercholesterolemia, cholesterol, lipoproteins, LDL, evolocumab, statin, cost-effectiveness, treatment outcome, quality of care, Bulgaria

## Abstract

**Background:** An elevated level of low-density lipoprotein cholesterol (LDL-C) constitutes one of the most important modifiable risk factors for cardiovascular disease (CVD). Individuals with heterozygous familial hypercholesterolaemia (HeFH) are particularly vulnerable to CVD events. The addition of evolocumab to statins has shown marked reductions in LDL-C levels. The objective of this analysis is to demonstrate the clinical and economic value of LDL-C lowering with evolocumab from the Bulgarian public health care perspective.

**Methods:** A disease-specific measure of health benefit was devised: Effectively treated patient-years (ETPYs) combine length of life with the likelihood of attaining best-practice recommendations on LDL-C lowering. “Effective treatment” was defined as a reduction in LDL-C levels of ≥50%. A Markov cohort state-transition model was adapted, considering a life-long treatment duration. Demographics, baseline characteristics and efficacy data were taken from the RUTHERFORD-2 trial. The model uses the relationship between LDL-C lowering and reduced CVD event rates observed in the meta-analyses conducted by the Cholesterol Treatment Trialists’ Collaboration. Outcomes and costs (from year 2015) were discounted at an annual rate of 5%. Sensitivity analyses were conducted to assess uncertainty surrounding the results.

**Results:** The total incremental costs of evolocumab added to statins versus statins alone are BGN 120,329 while adding 9.30 ETPYs over lifetime. These results imply an incremental cost per ETPY of BGN 12,937 (US$ 7,215; € 6,604). The use of evolocumab is associated with a relative reduction in the CVD event rate by 38% (18% per 1 mmol/L).

**Conclusions:** Adding evolocumab to statins may be considered cost-effective in light of an additional expense per patient-year gained in which individuals with HeFH receive effective treatment under the terms of international prevention guidelines. ETPYs are an intuitive and clinically meaningful measure of patient benefit that, in relation to costs, can support health care decision-making that considers quality of care.

## Background

An elevated level of low-density lipoprotein cholesterol (LDL-C) has been established as one of the most important modifiable risk factors for cardiovascular disease (CVD) [1,2], a medical condition that is the leading reason for hospitalisation in Bulgaria and accounts for nearly two-thirds of all-cause mortality [3]. If fatal, a CVD event shortens a person’s life by an estimated 17 years [4]. Non-fatal acute CVD events can lead to serious long-term consequences such as impaired quality of life and increased risk of subsequent CV events [5,6]. CVD is estimated to cost the Bulgarian society almost BGN 1.5 billion a year with approximately 54.4% being due to indirect costs – specifically, productivity losses and informal care costs [7].

The lowering of LDL-C levels is strongly associated with reductions in incidence of both fatal and non-fatal CVD events [8,9], making LDL-C a widely acknowledged therapeutic target [10]. Meta-analyses conducted by the Cholesterol Treatment Trialists’ Collaboration (CTTC) found that every 1 mmol/L reduction in LDL-C with statin therapy might lead to a 21–28% reduction in rates of any major CVD event [11–13].

Patients with heterozygous familial hypercholesterolaemia (HeFH), a hereditary condition that leads to life-long markedly raised LDL-C levels, are particularly vulnerable to CVD events; if untreated, the likelihood of premature coronary heart disease (CHD) is elevated about 20-fold [14]. The majority of these patients does not achieve an adequate reduction of LDL-C despite lipid-lowering therapy with the current standard of care (SoC), putting them at a risk for CVD that is 10 times greater than the risk for non-FH patients on comparable LDL-C lowering medication [15]. Proprotein convertase subtilisin/kexin type 9 (PCSK9) inhibition has emerged as a novel therapy for lowering LDL-C. Evolocumab (Repatha®, Amgen) is the first PCSK9 inhibitor that was shown to significantly reduce the incidence of major CVD events [16] and to regress or the stabilise atherosclerotic plaque [17]. The results of a trial in patients with HeFH showed that the addition of evolocumab to SoC (i.e. high-intensity statin therapy) led to a reduction in LDL-C levels of about 60% (RUTHERFORD-2 [18]).

Underpinned by evidence-based prevention guidelines, a disease-specific outcome measure – effectively treated patient-years (ETPYs) – was devised and subsequently used in an economic model that compared the addition of evolocumab to SoC versus SoC alone in patients with HeFH. This analysis sets out to demonstrate the clinical and economic value of LDL-C lowering with evolocumab from a Bulgarian public health care perspective.

## Methods

A previously published Markov cohort state transition model [19–21] was adapted, considering a Bulgarian payer perspective and a life-long treatment duration. The model employs annual cycles (with a half-cycle correction [22]) and was built using Microsoft Excel 2010 (Microsoft Corp., Redmond, WA). The 1-year cycle length is consistent with other economic evaluation studies in CVD [23].

### Outcome measure

Health technology assessment (HTA) in Bulgaria bases coverage decisions on a range of criteria that reflect societal preferences, including, among other considerations, disease severity and rarity, equity, already reimbursed therapies, the availability of funds, and quality of care. Judging by the Ordinance No. 9 that regulates the conditions and order for executing HTA [24], traditional cost-per-QALY evidence is not imperative to decision-making. We devised a disease-specific measure of health outcome that is underpinned by quality of care: effectively treated patient-years (ETPYs). ‘Effective treatment’ relates to best-practice recommendations advised by European (ESC/EAS) [25,26] and American (ACC/AHA) [27] guidelines of the management of dyslipidaemia to prevent CVD. These unanimously recommend that individuals with a high or very high CVD risk should receive treatment to achieve a reduction in LDL-C levels of 50% or more – a target currently attained by only 3.7% of Bulgarian HeFH patients [28,29]. ETPYs are thus calculated by multiplying the proportion of patients that attain a reduction in LDL-C levels of at least 50% by the predicted survival in each cycle. Since ETPYs, too, trace their origins to the epidemiologist’s measure of life-years, they are expected to highly correlate with QALYs. The incremental cost-effectiveness ratio (ICER) in this analysis is estimated as the incremental cost per incremental ETPY.

Additionally, the model evaluates life-years (LY), CVD deaths, non-fatal acute CVD event rates for myocardial infarction (MI), ischaemic stroke (IS) and heart failure (HF), and revascularisation rates.

### Model structure

**Figure 1.**
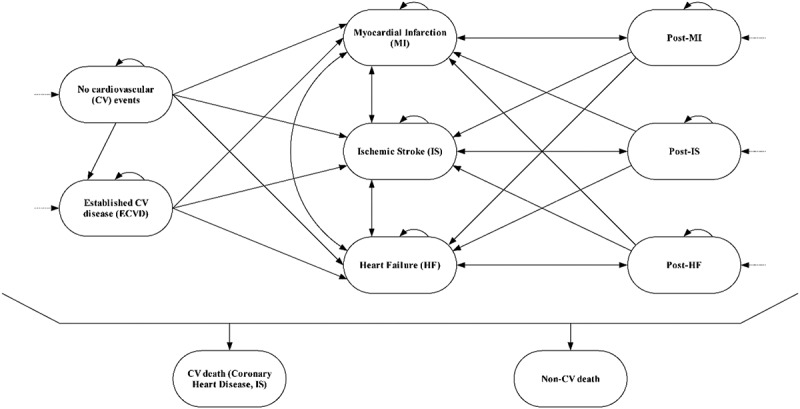
Evolocumab economic model structure. CHD, coronary heart disease; CVD, cardiovascular disease; ECVD, established CVD; IS, ischaemic stroke; HF, heart failure; MI, myocardial infarction

The mutually exclusive health states included in the model (Figure 1) are no CVD, established CVD (ECVD), myocardial infarction (MI), ischaemic stroke (IS), heart failure (HF), post-MI, post-IS, post-HF, coronary heart disease (CHD) death, IS death, and non-CVD death. The CVD events included under ECVD are transient ischaemic attack, peripheral vascular disease, stable angina, carotid stenosis, revascularisation in the absence of MI, and abdominal aortic aneurysm. Depending on their CVD event history, patients enter the model at the no CVD health state (61.7%), a post-event health state (14.9%), or the ECVD health state (23.4%). Revascularisation is included as a procedure, not as a separate health state, and the costs of revascularisation are included for a proportion of patients in the ECVD, MI, and post-MI health states according to published data [30,31].

Additionally, the model comprises thirteen composite health states created to retain memory of previous CVD events. The following assumptions for composite health states’ transition probabilities and costs apply: the composite’s risk to transition to a specific event equals the highest risk from the individual health states it contains. The highest of the costs for the individual health states is used as the cost for a composite health state (note that costs are not added up as this may lead to overestimation).

### Model inputs

The modelled population considers patients with HeFH and fasting LDL-C concentrations of 100 mg/dL or higher, with (38.3%) or without (61.7%) a history of CVD. The patient population had an average age of 51 years, was 42.3% female, and had a mean LDL-C level of 155 mg/dL. These data are from the RUTHERFORD-2 trial [18]. Table 1 shows the detailed baseline characteristics of this HeFH population.

**Table 1.** Patient demographics and baseline characteristics.

Characteristic	HeFH patients (N = 329)
Age, years (SD)	51.16 (12.60)
Female, %	42.25
Current smoking, %	15.81
Type 2 diabetes mellitus, %	7.29
Hypertensive therapy, %	32.52
Acetylsalicylic acid use, %	38.60
Systolic blood pressure, mmHg (SD)	125.73 (13.63)
Body mass index <20 kg/m^2^, %	1.82
Mean LDL-C, mg/dL (SD)	155.46 (44.93)
Mean Total-C, mg/dL (SD)	231.56 (48.66)
Mean HDL-C, mg/dL (SD)	51.13 (15.60)
Number of vascular beds*	1.21
Atrial fibrillation*, %	11.70
Previous CVD event (i.e. SP), %	38.30
Initial SP health state, %	
ECVD	61.11
Post-MI	25.40
Post-IS	2.38
Post-HF	0.79
Composite health states	10.32
Predicted 10-year risk of CVD events^†^	55%

SD, standard deviation; LDL-C, low-density lipoprotein cholesterol; HDL-C, high-density lipoprotein cholesterol; SP, secondary prevention; CVD, cardiovascular disease; ECVD, established CVD; IS, ischaemic stroke; HF, heart failure; HeFH, heterozygous familial hypercholesterolaemia; MI, myocardial infarction * Imputed based on the REACH Registry (Wilson et al. [32]) because of non-availability in RUTHERFORD-2 [18] ^†^ One or more fatal and non-fatal CVD event

### Baseline risk

Published risk-equations [32,33] are used on HeFH patient characteristics (Table 1) to estimate the baseline CVD risk. The Framingham risk equations [33] are used to predict the aggregate 10-year risk of CVD events in patients without a history of CVD. For patients with previous CVD events, the risk equation for ‘next cardiovascular event’ from the multinational REACH Registry [32] is employed to estimate the 20-month aggregate risk of recurrent CV events. As this analysis is carried out in the Bulgarian population, the coefficient for ‘Eastern Europe or Middle East’ was included in the risk equation [32]. The methodology described by Lothgren et al. [34] is used to remove any age effect on risk predictions.

Due to longer exposures to higher levels of LDL-C, HeFH patients have an elevated baseline CVD risk [14] compared with estimates based on the Framingham and REACH equations obtained from general hyperlipidaemic populations. To determine this level of baseline CVD risk, a literature search of publications reporting CVD risk in familial hypercholesterolaemia (FH) was conducted in which a total of 14 publications met inclusion criteria [35]. This review identified a Danish population-based study which included a direct comparison of FH and secondary prevention hyperlipidaemic populations reporting both fatal and non-fatal CVD events [15]. Compared with non-FH subjects off lipid-lowering therapy, the study reported event odds ratios (OR) (95% confidence intervals [CI]) of 13.2 (10.0–17.4) in FH subjects off therapy and 10.3 (7.8–13.8) in FH subjects on therapy, whilst adjusting for a number of risk factors. The reported ORs were used to calculate the rate ratios of CVD events in patients with FH compared with other hyperlipidaemic patients [36]. To mimic the real-world setting, the risks of treated and untreated groups were pooled to account for the mix of primary and secondary prevention patients. A rate ratio of 7.1 (5.7–8.7) was finally applied to the rates of events initially predicted to adjust for the increased risk in the modelled HeFH population [36]. The estimated 10-year risk of ≥1 CVD events is 55%.

### Efficacy and effectiveness

The predicted effectiveness of evolocumab on reducing CVD event rates is based on the relative LDL-C reduction observed in evolocumab’s phase 3 trial in HeFH patients, RUTHERFORD-2 [18]. Specifically, the model draws on the unpublished treatment difference between evolocumab (administered once every two weeks) and placebo in average percentage change in calculated LDL-C levels from baseline to the mean of weeks 10 and 12 (61.31%; 95% CI: 57.82–64.80%).

A treatment effect is applied to post-MI and ECVD patients requiring revascularisation, however not for acute MI patients, as the revascularisation rate is mediated by the reduced incidence of acute MI. Furthermore, no treatment effect is applied for HF as no impact of LDL-C lowering on the HF event rate has been reported in the CTTC meta-analyses [11–13].

The proportion of statin-treated HeFH patients that attain a reduction in LDL-C levels of ≥50% when given added placebo or added evolocumab is 1.96% (95% CI: 0.05–10.45%) and 79.25% (95% CI: 70.28–86.51%), respectively. It was calculated using unpublished patient-level data in those with LDL-C levels >100 mg/dL from RUTHERFORD-2. Due to the consistent effect of evolocumab, no statistically significant difference in proportion of patients being effectively treated is seen between the prespecified subgroups of the trial. Therefore, the overall percentage of effectively treated patients is used to calculate the primary outcome measure of this analysis: the effectively treated patient-years (ETPYs).

### Relationship between LDL-C lowering and reduced CVD event rates

Lowering LDL-C levels has been shown to reduce the risk of CVD events in several interventional and epidemiologic studies [11–13,37–39]; that holds for both statin [40,41] and non-statin therapies [42,43]. The evolocumab CV outcomes trial, Further Cardiovascular Outcomes Research with PCSK9 Inhibition in Subjects With Elevated Risk (FOURIER) [16], demonstrated that the addition of evolocumab to SoC is associated with a reduced incidence of major CVD events.

The model employs the relationship between statin-generated LDL-C lowering and reduced CVD event rates from the CTTC meta-analyses [11–13]. These provide robust, event-specific effect estimates that have been extensively used in previous economic evaluations of lipid-lowering therapies and are considered the gold standard for estimating treatment effect associated with LDL-C reduction. Compared to CTTC data, treatment with evolocumab has very similar effects on the risk of major CVD events [16]. In light of the current body of evidence, the European Atherosclerosis Society (EAS) deems PCSK9 inhibitors as equivalent to statins in terms of their effects on the risk of CV events per unit decrease in LDL-C [44].

Table 2 details the rate ratios per 1 mmol/L of LDL-C reduction that are applied to the predicted event rates at baseline. They represent changes in CVD event rates associated with an absolute reduction in LDL-C levels observed in the CTTC meta-analyses.

**Table 2.** CVD event rate ratio per 1 mmol/L (38.67 mg/dL) of LDL-C reduction.

Event	Rate Ratio (99% CI)	Source
ECVD	0.71 (0.58–0.87)	Assumed to be equivalent to MI
MI	0.71 (0.58–0.87)	CTTC [11] Figure 2 (more vs less statin)
IS	0.69 (0.50–0.95)	CTTC [11] Figure 2 (more vs less statin)
CHD death	0.80 (0.76–0.85)	CTTC [12] Webfigure 3 and 8 (overall)
IS death	1.00	Assumed equal to 1 because of statistical non-significance (1.04; 95% CI: 0.77–1.41) [11]
Revascularisation*	0.66 (0.60–0.73)^†^	CTTC [11] Figure 2 (more vs less statin)

CI, confidence interval; ECVD, established cardiovascular disease; IS, ischaemic stroke; CHD, coronary heart disease; CTTC, Cholesterol Treatment Trialists’ Collaboration; MI, myocardial infarction * Defined as percutaneous coronary intervention (PCI) or coronary artery bypass grafting (CABG) † Denoting a 95% CI

**Figure 2.**
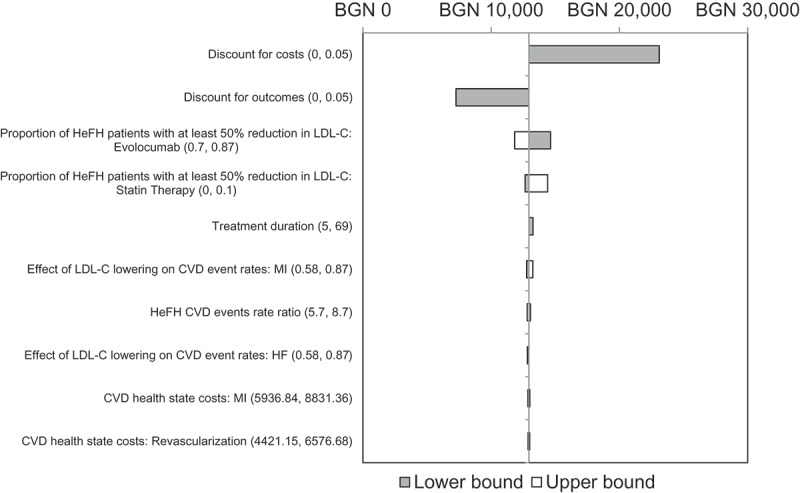
Univariate sensitivity analyses. CVD, cardiovascular disease; ECVD, established CVD; HeFH, heterozygous familial hypercholesterolaemia; LDL-C, low-density lipoprotein cholesterol; MI, myocardial infarction

The rate of CVD events after treatment is given by the following formula:

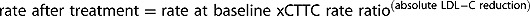

Based on this, the modified CVD event risk (i.e. the consequent impact of treatment on occurrence of CVD events and survival) was derived.

### Event distribution

Based on CPRD/HES longitudinal data, multinomial logistic regression models were used to estimate the proportion of cardiovascular events (MI, IS, HF, CV death) conditional on prior events [45]. In addition, for patients who had a CV death, logistic regression was used to predict whether the event was cerebrovascular or otherwise.

### Mortality

CVD mortality was predicted as a consequence of incident CVD events. Mortality from other causes than CVD was assumed to be the same as that of the general population, taken from life tables by age and gender published by the National Statistical Institute Bulgaria [46]. It was derived separately by subtracting the components of ischaemic heart diseases (ICD I20-I25) and cerebrovascular diseases (ICD I60-I69) from the all-cause mortality.

### Costs

The annual cost of evolocumab corresponds to the basic reimbursed price in Bulgaria after applying a mandatory payback to the National Health Insurance Fund, NZOK, according to local legislation [47]. Annual costs for currently reimbursed high-intensity statins in Bulgaria (atorvastatin 40–80 mg; rosuvastatin 20–40 mg [48]) are calculated using a weighted average based on their market share. Market shares were derived from IMS proprietary data [49]. To level the playing field, a 100% reimbursement of statins has been assumed for the target population.

Medical costs associated with CVD health states (Table 3) were obtained from the National Health Insurance Fund, NZOK [47,50]. Resource use estimates relevant to practice patterns in Bulgaria were derived from expert consultation with Prof. Ivo Petrov, national consultant of cardiology. First-year acute and short-term costs as well as post-event costs for subsequent years were considered. Indirect costs irrelevant to the Bulgarian public payer were not included in this analysis.

**Table 3.** CVD event costs per patient per year.

	Annual direct cost (BGN)		
Modelled health state	Acute (year 1)	Post-event (subsequent years)	Source
No CVD	0.00	–	Assumption
ECVD*	–	582.05	[47,50]
MI	7,384.10	582.05	[47,50]
IS	4,154.84	223.03	[47,50]
HF	2,569.28	2,564.61	[47,50]
CHD death	5,171.50	–	[47,50]
28.3% Fatal UA	5,270.00		
56.6% Fatal MI	6,380.00		
15% Fatal HF	420.00		
IS death	8,095.00	–	[47,50]
Non-CVD death	0.00	–	Assumption
Revascularisation	5,498.92	–	[47,50]
78.5% PCI	4,238.00		
21.5% CABG	10,100.00		

CABG, coronary artery bypass grafting; CVD, cardiovascular disease; ECVD, established CVD; IS, ischaemic stroke; HF, heart failure; CHD, coronary heart disease; UA, unstable angina; MI, myocardial infarction; HF, heart failure; MI, myocardial infarction; PCI, percutaneous coronary intervention * Assumed to be equivalent to the costs attributed to subsequent years of MI (BGN 582.05)

RUTHERFORD-2 [18] did not find any notable differences in the adverse event profiles between evolocumab-treated and placebo-treated patients. Therefore, incidence and costs associated with adverse events are not included in the economic evaluation.

### Base-case analysis

Health outcomes in this cost-effectiveness analysis were summarised in the form of effectively treated patient-years (ETPYs), combining length of life with a quality of care component. LYGs, CVD event and revascularisation rates were also presented. All outcomes and costs were discounted at a rate of 5%, according to Bulgarian guidelines [24].

### Sensitivity analyses

Both univariate deterministic and multivariate probabilistic sensitivity analyses were used to assess uncertainty surrounding the incremental cost per ETPY gained. For the one-way sensitivity analyses, the baseline CVD risk adjustment, the relative reduction in LDL-C observed among evolocumab-treated patients, the CTTC CVD event rate ratios, and the proportion of patients attaining a reduction in LDL-C levels of ≥50% were changed to the lower and upper bound of their 95% CIs. A standard error of 10% of the mean values was assumed to calculate the 95% CIs for health state costs. The base-case treatment duration (lifetime) and discount rate on costs and outcomes (5%) were lowered to 5 years and 0%, respectively.

Probabilistic sensitivity analysis was additionally conducted in order to fully examine the combined effect of parameter uncertainty on the base-case result. Appropriate probability distributions following Briggs et al. [51] (*beta*, relative reduction in LDL-C and proportion of patients effectively treated; *gamma*, health state costs; *lognormal*, CTTC rate ratios and baseline CVD risk adjustment) were assigned to model parameters based on their respective means and standard errors. Values for parameters were then sampled by Monte Carlo simulation with 1,000 iterations in each loop.

## Results

The total incremental costs of evolocumab added to SoC (high-intensity statins) versus SoC alone are BGN 120,329. Meanwhile, evolocumab-treated patients also gain 9.30 effectively treated patient-years over lifetime. These results imply an incremental cost per ETPY of BGN 12,937 (US$ 7,215; € 6,604). The use of evolocumab is associated with a relative reduction in the CVD event rate by 38% (18% per 1 mmol/L); non-fatal acute CVD events decrease by 44% and a 17% reduction in the CVD death rate is estimated (Table 4). The initial cross-sectional HeFH rate ratio of 7.1 translated into HeFH patients predicted to have 5.1 times more events over a lifetime horizon than non-HeFH patients with a similar risk profile.

**Table 4.** Predicted CVD event rates, ETPYs and costs.

	Evolocumab + SoC	SoC alone	Increment (∆)
Total LYs*	12.07	11.15	0.93
Total ETPYs*	**9.52**	**0.22**	**9.30**
CVD events	1.81	2.92	−1.11
MI	0.72	1.63	−0.92
IS	0.11	0.26	−0.16
HF	0.44	0.36	0.08
Fatal CVD	0.54	0.65	−0.11
Revascularisation	0.65	1.65	−1.00
**Costs (BGN)**			
**Total cost**	**139,741**	**19,412**	**120,329**
Medication	128,592	1,432	127,160
Non-fatal acute CVD events	3,239	7,088	−3,849
Fatal CVD events	1,201	1,545	−344
Revascularisation	1,866	4,862	−2,995
Post-event	3,480	3,381	99
**ICER (BGN/ETPY gained)**			**12,937**
**Probabilistic sensitivity analysis results**			
Mean ETPY	9.51	0.22	9.29
Mean cost (BGN)	139,853	19,400	120,454
**ICER (BGN/ETPY gained)**			**12,963**

SoC, standard of care; LYs, life-years; ETPYs, effectively treated patient-years; CVD, cardiovascular disease; IS, ischaemic stroke; HF, heart failure; ICER, incremental cost-effectiveness ratio; MI, myocardial infarction

* Discounted

### Sensitivity analyses

Figure 2 presents a tornado diagram containing the 10 parameters which have the largest effect on the base-case ICER. The results indicate that the ICER is mainly sensitive to changes in the discount rate on costs and outcomes. Reducing the discount rate applied to costs to 0% causes the largest increase in ICER, whereas an equivalent change of the discount rate applied to ETPYs considerably lowers it. For the proportions of patients attaining a reduction of LDL-C of at least 50%, the ICER does not exceed the range of BGN 11,827–14,632 per ETPY gained. Overall, the ICERs are robust to changes in efficacy and cost parameters. Table 4 includes the results of the probabilistic sensitivity analysis.

## Discussion

The presented economic evaluation in the Bulgarian context shows that, in HeFH patients, the use of evolocumab added to SoC compared with SoC alone results in an ICER of BGN 12,937 (US$ 7,215; € 6,604) per ETPY gained. Sensitivity analyses confirm the robustness of the model results.

Effectively treated patient-years (ETPYs) is an intuitive and clinically meaningful measure of patient benefit, combining length of life with the likelihood of attaining internationally acknowledged best-practice recommendations on LDL-C lowering [25–27] aimed at reducing CVD events. Quality-of-care-adjusted survival can help decision makers to evaluate the overall outcome and value of a new technology compared with well-defined existing practice. The concept of effectively treated patients has been used in different indications [52,53] to support health care decision-making and policy. Based on the analysis presented herein, the Committee on Health Technology Assessment recommended inclusion of evolocumab within the Bulgarian national drug formulary [54].

The proposed outcome measure is most useful for deliberative HTA decision-making, not subject to the cost-per-QALY paradigm, that recognises and considers process-oriented factors of value, specifically quality of care. Cost-per-QALY evidence is immaterial in Bulgaria for various reasons. First, funding decisions in the country’s decentralised health care system are usually made within a therapeutic area and not across differing indications, thereby reducing considerably the relevance of the quality-adjusted life year (QALY) as a common measure of benefit. Second, population-specific quality-of-life evidence is sparse, which in effect hinders the very calculation of QALYs. Third, using the cost-per-QALY metric in Bulgaria would be unlikely to contribute to its prime objective: promoting allocative efficiency. Policy-makers commonly compare a technology’s incremental cost-per-QALY to a predefined threshold in order to assess its value for money. Under a single fixed health care budget, this threshold conceptually represents the opportunity costs of health care spending, i.e. the alternative health investments that must be sacrificed as a consequence of the additional funds needed to pay for any new cost-increasing technology. More often than not, the magnitude of this threshold has little or no empirical foundation; in Bulgaria, no mandatory consensus standard exists for it. On the other hand, the often adduced WHO-CHOICE threshold of 1 to 3 times GDP per capita [55], also cited by the National Council of Pricing an Reimbursement [56], has several well-acknowledged shortcomings [57,58] and is therefore contentious. Invoking thresholds that do not reflect the opportunity costs of a particular health care system is not expedient to advance the health-related well-being of the population it serves. Apart from that, estimating the true value of the threshold that represents the health opportunity costs using empirical research is challenging as data demands may be excessive [59]. To our knowledge, no such attempts have yet been made, nor are intended to be made in Bulgaria.

Over two-thirds of the Bulgarian population think the overall quality of health care in their country is bad [60] and 5.6% self-declare unmet needs for health care services due in part to inadequate funding [61]. Although the latter figure remains higher than the European average (3.6%), it has appreciably fallen in the 4 years leading to 2014, the last year for which data exist. The utilisation of evolocumab in HeFH patients at increased risk for CVD and unable to control LDL-C levels with established statin therapy provides an opportunity to contribute to the continuation of this positive trend.

There are a number of limitations in this analysis. The effectiveness of evolocumab on reducing CVD event rates is predicated upon short-term LDL-C lowering [18], assumed to remain constant over a life-long treatment duration. This is supported by the sustained reductions in LDL-C observed over 4 years of evolocumab treatment in the open-label, randomised extension study OSLER-1 [62], which also included HeFH patients. Furthermore, if the levels of persistence with and adherence to evolocumab therapy in actual clinical practice differ from those in the RUTHERFORD-2 trial, the costs and efficacy of evolocumab will be impacted. Finally, the results of this analysis are not generalisable to populations other than HeFH with a similar risk profile as described herein.

## Conclusions

When used in HeFH patients unable to control LDL-C levels with high-intensity statin therapy and who therefore remain at high risk of CVD, the addition of evolocumab may be considered cost-effective in light of an additional expense of BGN 12,846 (€ 6,559) per patient-year gained in which individuals receive effective treatment under the terms of international prevention guidelines.

## List of Abbreviations

ACC American College of Cardiology

AHA American Heart Association

BGN ISO 4217 currency code of the Bulgarian lev, the currency of Bulgaria

CABG Coronary artery bypass graft

CHD Coronary heart disease

CI Confidence inteval

CPRD Clinical Practice Research Datalink

CTTC Cholesterol Treatment Trialists’ Collaboration

CV Cardiovascular

CVD Cardiovascular disease

EAS European Atherosclerosis Society

ECVD Established cardiovascular disease

ESC European Society of Cardiology

ETPY Effectively treated patient-year

FH Familial hypercholesterolaemia

FOURIER Further Cardiovascular Outcomes Research with PCSK9 Inhibition in Subjects With Elevated Risk

GDP Gross domestic product

HDL-C High-density lipoprotein cholesterol

HeFH Heterozygous familial hypercholesterolaemia

HES Hospital Episode Statistics

HF Heart failure

HTA Health technology assessment

ICD International Classification of Diseases

ICER Incremental cost-effectiveness ratio

IS Ischaemic stroke

LDL-C Low-density lipoprotein cholesterol

LY Life years

Mg/dL Milligrams per decilitre

MI Myocardial infarction

NZOK National Health Insurance Fund

ONS Office for National Statistics

OR Odds ratio

PCI Percutaneous coronary intervention

PCSK9 Proprotein convertase subtilisin/kexin type 9

QALY Quality-adjusted life year

SD Standard deviation

SoC Standard of care

SP Secondary prevention

UA Unstable angina
